# Associations of Estrogen and Testosterone With Insulin Resistance in Pre- and Postmenopausal Women With and Without Hormone Therapy

**DOI:** 10.5812/ijem.5333

**Published:** 2013-04-01

**Authors:** Sumika Matsui, Toshiyuki Yasui, Anna Tani, Kotaro Kunimi, Hirokazu Uemura, Satoshi Yamamoto, Akira Kuwahara, Toshiya Matsuzaki, Minoru Irahara

**Affiliations:** 1 Department of Obstetrics and Gynecology, Institute of Health Biosciences, The University of Tokushima Graduate School, Tokushima, Japan, Tokushima 770-8503, Japan; 2 Department of Reproductive Technology, Institute of Health Biosciences, The University of Tokushima Graduate School, Tokushima, Japan; 3 Department of Preventive Medicine, Institute of Health Biosciences, The University of Tokushima Graduate School, Tokushima, Japan

**Keywords:** Estradiol, Testosterone, Sex Hormone-Binding Globulin, HOMA-IR

## Abstract

**Background:**

Estrogen deficiency due to natural menopause or surgical menopause has been suggested to have an adverse effect on insulin resistance. Testosterone and sex hormone–binding globulin (SHBG) as well as estrogen are also associated with insulin resistance in women. However, to date, the associations of estradiol, testosterone and SHBG with insulin resistance according to estrogen level have not been clarified.

**Objectives:**

We examined the associations of estradiol, testosterone and SHBG with insulin resistance in pre- and in postmenopausal women and postmenopausal women who had received hormone therapy to clarify whether the associations differ depending on the estrogen status.

**Patients and Methods:**

Twenty premenopausal women and thirty-two postmenopausal women were enrolled in this study. Fifteen postmenopausal women received oral conjugated equine estrogen (CEE) (0.625 mg) everyday for 12 months. Serum levels of estradiol, testosterone, SHBG and insulin and plasma levels of glucose were measured.

**Results:**

Serum estradiol levels tended to have a negative correlation with homeostasis model assessment of insulin resistance (HOMA-IR) in premenopausal women but not in postmenopausal women. On the other hand, free testosterone levels tended to have a positive correlation with HOMA-IR in postmenopausal women but not in premenopausal women. Serum SHBG levels showed significant negative correlations with HOMA-IR in both pre- and postmenopausal women. SHBG level was significantly increased, free testosterone level was significantly decreased and HOMA-IR was significantly decreased at 12 months after CEE administration. However, there were no significant correlations of changes between estradiol, SHBG or free testosterone and HOMA-IR.

**Conclusions:**

The associations of sex steroid hormones with insulin resistance are different depending on the estrogen status.

## 1. Background

Estrogen deficiency, due to natural menopause or surgical menopause, has been suggested to have an effect on insulin resistance (1-3). Homeostasis model assessment of insulin resistance (HOMA-IR) as a surrogate marker of insulin resistance based on measurement of fasting plasma glucose and insulin, has been widely used in clinical practice. It has been reported that HOMA-IR in postmenopausal women was significantly higher than the one in premenopausal women (4, 5). However, several studies have shown that estrogen deficiency did not affect insulin resistance (6-9). Davis et al. suggested that there was no independent relationship between estradiol and HOMA-IR in postmenopausal women (10). Testosterone and sex hormone-binding globulin (SHBG) as well as estrogen are also associated with insulin resistance in women. In premenopausal women with polycystic ovary syndrome, it has been reported that testosterone and SHBG were associated with insulin resistance (11, 12). In postmenopausal women, it has been suggested that a high testosterone level has an unfavorable effect on insulin resistance (13-16). Sutton et al. reported that a high free androgen index was positively associated with HOMA-IR (13). In addition, it has been reported that SHBG level was inversely associated with HOMA-IR (17) and that a low SHBG level was associated with the risk of onset of type 2 diabetes in postmenopausal women (18). However, to date, the associations of estradiol, testosterone and SHBG with insulin resistance, according to estrogen level, have not been clarified. In the present study, we examined the associations of estradiol, testosterone and SHBG with insulin resistance in premenopausal women, postmenopausal women and postmenopausal women who had received hormone therapy to clarify whether the associations differred depending on the estrogen status.

## 2. Patients and Methods

## 2.1. Patients

The subjects of this study were recruited from patients visiting the outpatient clinic of the Department of Obstetrics and Gynecology, Tokushima University Hospital. Twenty premenopausal women and 32 postmenopausal women including 15 postmenopausal women who had received hormone therapy, were enrolled in this study. The premenopausal women included 15 women who had a recent history of regular menstruation (25–35 days per cycle) and no hysterectomy or bilateral oophorectomy, and 5 women who had experienced alterations in menstrual frequency and/or flow in the 12 months preceding entry into the study. Postmenopausal status was confirmed by follicle-stimulating hormone (FSH) concentration ≥ 40 mIU/mL and estradiol concentration ≤ 20 pg/mL in women with no natural menstruation for at least 1 year or, women who had undergone bilateral oophorectomy. Fifteen postmenopausal women received oral conjugated equine estrogen (CEE) (0.625 mg) everyday for 12 months. Oral medroxyprogesterone acetate (MPA) (2.5 mg) was continuously administered for endometrial protection except for nine subjects who underwent hysterectomy．Before recruitment in the study, patients underwent gynecological and biochemical examinations that included bimanual examination and transvaginal ultrasonography. Reviews of medical histories and results of physical examinations and blood chemistry tests, showed that all of the women were in good health. Exclusion criteria in the study were a history of any cardiovascular disease, hormone-dependent malignancy or breast cancer, venous thromboembolic disease, diabetes mellitus, renal dysfunction, liver disease, hypertension, and use of lipid-lowering drugs. Women who had received hormone therapy in the past were not included in the study. None of the subjects had taken any medication known to influence glucose metabolism for at least one year. Venous blood samples for measurements of insulin and hormones were drawn into BD vacutainer tubes (Becton, Dickinson and Company, Franklin Lakes, New Jersey), and blood samples for measurement of glucose were drawn into Venoject II tubes containing NaF, EDTA-2Na and heparin sodium (Terumo Corporation, Tokyo, Japan) between 8 AM and 10 AM after 12-hour fasting before and at 12 months after commencement of the study. Blood samples were taken from premenopausal women during the follicular phase. Blood samples obtained were frozen at -70℃ until used for analysis. Informed consent for participation in this study was obtained from each woman. The Ethics Committee of Tokushima University Hospital approved the study.

## 2.2. Measurement of Serum Concentrations of Estradiol and FSH

Serum estradiol concentration was measured by a two-site immunoenzymometric assay using a commercially available kit (TOSOH Co., Tokyo, Japan, catalog number: 13A2X00174000006). The intra- and inter-assay coefficients of variation (CV) ranged from 4 to 9% and 6 to 9%, respectively. The range of measurement was 25 to 3000 pg/mL and the sensitivity of the assay was 25 pg/mL. Serum FSH concentration was measured by an IRMA using a commercially available kit (TFB Co., Tokyo, Japan, catalog number: 16300AMZ00551000). The intra- and inter-assay CVs ranged from 3 to 4% and 3 to 4%, respectively. The range of measurement was 0.5 to 200 mIU/mL and the sensitivity of the assay was 0.5 mIU/mL.

## 2.3. Measurement of Serum Testosterone and SHBG

Serum total testosterone concentration was measured by an electrochemiluminescence immunoassay using a commercially available kit (Roche Diagnostics, Basel, Switzerland, catalog number: 13A2X00206000132). The intra- and inter-assay CVs ranged from 2.2 to 3.2% and 3.6 to 4.6%, respectively. The sensitivity of the assay was 0.05 ng/mL. Serum SHBG concentration was measured by a solid-phase immunoradiometric assay (Siemens Medical Solutions Diagnostics, Los Angeles, CA, USA, catalog number: RKSH1). The intra- and inter-assay CVs ranged from 2.8 to 5.3% and 7.9 to 8.5%, respectively, and the sensitivity of the assay was 1 nmol/L. Serum free testosterone was calculated using total testosterone, albumin and SHBG by a previously described method (19).

## 2.4. Measurement of Concentrations of Glucose and Insulin

Plasma glucose level was measured by using the glucose oxidase method on an Automated Gluose Analyzer GA04, which is a method for measurement of plasma glucose by reacting the sample with glucose oxidase (A&T, Kanagawa, Japan, catalog number: 21300BZZ00323000). The intra- and inter-assay CVs ranged from 0.8 to 1.3% and 0.6 to 1.3%, respectively. Serum insulin level was measured by using an enzyme immunoassay on AIA2000 (TOSOH Co., Tokyo, Japan, catalog number: 13A2X00174000010). The intra- and inter-assay CVs ranged from 1.1 to 3.2% and 1.9 to 3.3%, respectively, and the sensitivity of the assay was 1.0 μU/mL. Insulin resistance was evaluated with HOMA-IR, which was calculated for all subjects by the following formula: fasting serum insulin (μU/mL) x fasting plasma glucose (mg/dL)/405.

## 2.5. Statistical Analysis

Baseline characteristics and serum levels of sex steroid hormones, glucose and insulin are presented as medians with 25th and 75th percentile ranges, and the significance of differences between those values in the two groups was evaluated by the Mann-Whitney U test. Baseline and follow-up levels of hormones, glucose and insulin were compared across the same group by the non-parametric Wilcoxon signed-ranks test. We set the basal values as 100% and calculated the percentage changes. Correlations between hormones and HOMA-IR, and between percentage changes from baseline levels of hormones and HOMA-IR, were determined by using Spearman’s rank order analysis and partial rank order analysis. All statistical analyses were performed using Statview (SAS Institute Inc.). P values less than 0.05 were considered to be statistically significant. In previous studies, values below the detection limit were defined as half of the detection limit in analyses (20, 21). We performed statistical analysis by using half of the value of sensitivity when the values were below the sensitivity limit.

## 3. Results

## 3.1. Levels of Hormones, SHBG and HOMA-IR in Pre- and Postmenopausal Women 

Baseline characteristics in pre- and postmenopausal women are presented in Table 1. Serum estradiol levels in postmenopausal women were significantly lower than those in premenopausal women. There was no significant difference between levels of testosterone or SHBG in pre- and postmenopausal women. Plasma glucose levels in postmenopausal women were significantly higher than those in premenopausal women, but there were no significant differences in insulin levels and HOMA-IR.

**Table 1.** Baseline Characteristics and Serum Levels of Hormones, SHBG and HOMA-IR in Premenopausal Women and Postmenopausal Women

	Premenopausal Women (n=20), median (25-75th percentile ranges)	Postmenopausal Women (n=32), median (25-75th percentile ranges)	P value
**Age, y** **^a^**	42.00 (40.00-44.25)	50.00 (46.75-53.00)	< 0.001
**BMI, kg/m** **^2^**	20.25 (19.56-23.43)	21.13 (19.85-23.13)	0.499
**FSH, mIU/m** **L**	7.250 (4.800-10.30)	93.10 (85.63-131.1)	< 0.001
**Estradiol, pg/m** **L**	96.50 (69.00-154.0)	12.50 (12.50-18.00)	< 0.001
**T, ng/m** **L**	0.210 (0.098-0.293)	0.180 (0.148-0.285)	0.992
**Free ** **T, ng/d** **L**	0.217 (0.076-0.278)	0.165 (0.130-0.370)	0.137
**SHBG, ** **nmol/** **L**	88.80 (67.20-113.5)	81.65 (59.43-95.88)	0.156
**Glucose, mg/d** **L**	86.00 (78.80-95.80)	96.50 (90.00-100.3)	0.016
**Insulin, μU/m** **L**	7.635 (4.898-12.75)	5.310 (2.918-12.55)	0.125
**HOMA-IR**	1.659 (0.934-3.139)	1.276 (0.661-2.996)	0.284

^a^Abbreviations: BMI, body mass index; FSH, follicle-stimulating hormone; HOMA-IR, homeostasis model assessment of insulin resistance; SHBG, sex hormone-binding globulin; T, testosterone; y, year.

## 3.2. Associations of HOMA-IR With Hormones and SHBG in Pre- and Postmenopausal Women 

As can be seen in Table 2, BMI was positively correlated with HOMA-IR in postmenopausal women, but there was no significant correlation in premenopausal women. Serum estradiol levels tended to have a negative correlation with HOMA-IR in premenopausal women but not in postmenopausal women. On the other hand, free testosterone levels tended to have a positive correlation with HOMA-IR in postmenopausal women but not in premenopausal women. This tendency for a positive correlation continued after adjustment for age and BMI. Serum SHBG levels showed significant negative correlations with HOMA-IR in both pre- and postmenopausal women, and this correlation was still significant after adjustment for age and BMI in premenopausal women. Serum SHBG levels showed negative tendency to correlate with HOMA-IR in postmenopausal women that remained after adjustment for age and BMI.

**Table 2.** Correlations of HOMA-IR With Age, BMI, Hormones and SHBG (M1) and Correlations After Adjustment for Age and BMI (M2) in Premenopausal Women and Postmenopausal Women

		Premenopausal Women (n = 20)		Postmenopausal Women (n = 32)	
		M1	M2	M1	M2
**Age **	r	0.431	-	-0.297	-
	p	0.058	-	0.099	-
**BMI** **^ a^**	r	0.246	-	0.495	-
	p	0.326	-	0.010	-
**Estradiol **	r	-0.414	-0.459	-0.005	-0.034
	p	0.070	0.079	0.979	0.873
**T **	r	-0.265	-0.272	0.210	0.313
	p	0.259	0.309	0.250	0.128
**Free T**	r	-0.057	0.009	0.307	0.364
	p	0.811	0.975	0.088	0.074
**SHBG **	r	-0.522	-0.577	-0.453	-0.39
	p	0.018	0.019	0.009	0.054

^a^Abbreviations: BMI, body mass index; HOMA-IR, homeostasis model assessment of insulin resistance; SHBG, sex hormone-binding globulin; T, testosterone.

## 3.3. Changes in Serum Concentrations of SHBG, Testosterone and HOMA-IR in Women Who Received Hormone Therapy

Median age and BMI of the subjects who received hormone therapy were 51.0 years and 20.6 kg/m2, respectively. All participants originally enrolled in the study, completed the 12-month study without severe adverse effects. BMI was not changed significantly at 12 months after hormone therapy. As can be seen in Table 3, serum SHBG levels were significantly increased and free testosterone levels were significantly decreased at 12 months after hormone therapy. Serum insulin levels tended to be lower and HOMA-IR was significantly decreased, while plasma glucose levels were not changed.

**Table 3.** Serum Levels of Hormones, SHBG and HOMA-IR at Baseline and After 12 Months in Women Receiving Oral CEE Administration

	Baseline (n = 15), median (25-75th percentile ranges)	After 12 Months (n = 15), median (25-75th percentile ranges)	P value
**Estradiol, pg/m** **L**	14.75 (12.50-18.50)	32.20 (25.00-54.00)	0.012
**FSH, mIU/m** **L**	93.00 (91.10-131.6)	76.30 (55.45-137.1)	0.056
**Testosterone, ng/m** **L**	0.170 (0.145-0.230)	0.230 (0.175-0.280)	0.506
**Free T, ng/d** **L**	0.156 (0.116-0.231)	0.135 (0.126-0.165)	0.024
**SHBG, nmol/** **L**	91.60 (78.90-99.70)	121.5 (97.53-150.5)	0.015
**Glucose, mg/d** **L**	91.00 (87.50-98.50)	93.00 (86.00-99.50)	0.916
**Insulin, μU/m** **L**	4.010 (2.685-9.845)	3.140 (2.275-5.555)	0.078
**HOMA-IR,**	0.947 (0.628-2.156)	0.644 (0.532-1.212)	0.047

## 3.4. Associations of HOMA-IR with Hormones and SHBG in Postmenopausal Women Who Received Hormone Therapy, and Associations Between Changes in Estradiol, Free Testosterone and SHBG and Decreases in Insulin and HOMA-IR

BMI showed a significant positive correlation with HOMA-IR significantly, but there were no significant correlations of HOMA-IR with estradiol, testosterone, free testosterone and SHBG at 12 months after hormone therapy. In addition, we examined the correlations between percentage changes in estradiol, free testosterone and SHBG and those in insulin and HOMA-IR to determine whether increases in estradiol and SHBG and decrease in free testosterone are associated with decreases in insulin and HOMA-IR. However, there were no significant correlations between the percentage changes (data not shown).

## 4. Discussion

We found that the associations of estradiol, testosterone and SHBG with insulin resistance were different in premenopausal women, postmenopausal women and postmenopausal women who had received hormone therapy. In the present study, estradiol level tended to have a negative correlation with HOMA-IR in premenopausal women but not in postmenopausal women. On the other hand, free testosterone level tended to have a positive correlation with HOMA-IR in postmenopausal women but not in premenopausal women after adjustment for age and BMI. It is possible that estrogen protects against insulin resistance and that the influence of testosterone on insulin resistance is weak in the presence of estrogen, such as that in the premenopausal stage. However, testosterone may be involved in insulin resistance in a state of estrogen deficiency. Golden et al. reported that bioavailable testosterone levels were associated with insulin resistance in postmenopausal women (17). In pre- and perimenopausal women, it has been reported that higher free testosterone was related with higher insulin, glucose and HOMA-IR after adjustment for BMI and that estradiol was not correlated with those markers of glucose metabolism (13). The difference between the results of our study and those of the previous study may be due to differences in background characteristics, such as age and BMI of the subjects, and difference in serum estradiol level. Interest has recently been shown in biological functions of SHBG associated with insulin resistance beyond its role as simply a transporter of sex steroid hormones (17). However, the mechanism regarding the effect of SHBG on insulin resistance has not been clarified. We showed that SHBG levels were inversely correlated with HOMA-IR in both pre- and postmenopausal women. It is thought that sex steroid hormones regulate the hepatic production of SHBG (22). However, Davis et al. reported that the contribution of SHBG to HOMA-IR was not dependent on endogeneous estrogen or androgen levels in postmenopausal women, suggesting that the independent inverse relationship between SHBG and insulin resistance is related to the regulation of hepatic SHBG synthesis (10). SHBG may be a key factor related to insulin resistance independent of estradiol and testosterone. In the present study, we showed that BMI was positively correlated with HOMA-IR in postmenopausal women but not in premenopausal women. It has been reported that high BMI, such as that in overweight or obese persons, was associated with insulin resistance (23), but estrogen may have a protective effect against insulin resistance in premenopausal women. The association of BMI with HOMA-IR might be easily influenced by the presence of estrogen in women who are not overweight or obese since overweight and obese women were not included in our study. We did not find a correlation between estradiol level and HOMA-IR at 12 months after hormone therapy, and the correlations between percentage changes in estradiol level and those in HOMA-IR. Circulating estradiol level in postmenopausal women receiving estrogen replacement therapy was lower than that in premenopausal women. Estradiol level that is comparable to that in premenopausal women may be needed for improvement of insulin sensitivity by hormone therapy in postmenopausal women. In addition, we used CEE, not 17β-estradiol, for hormone therapy. The effect of CEE on insulin resistance may be different from the effect of 17β-estradiol. It has been reported that the effects of oral CEE and oral 17β-estradiol on insulin resistance were different (24). It has been shown in the Women’s Health Initiative trial that hormone therapy with CEE plus MPA reduced the incidence of new-onset diabetes (25). In addition, administration of oral CEE induced an increase in SHBG and a decrease in free testosterone (26). Christodoulakos et al. reported that these hormonal changes during administration of CEE and MPA were associated with insulin resistance (24). Our results regarding changes in free testosterone, SHBG and HOMA-IR during oral CEE plus MPA administration were in line with the results of previous studies (24, 26). However, levels of free testosterone and SHBG were not correlated with HOMA-IR in women at 12 months after hormone therapy, and percentage changes in levels of free testosterone and SHBG due to oral CEE administration were not correlated with percentage changes in HOMA-IR during hormone therapy. The associations of testosterone with insulin resistance in women receiving estrogen replacement therapy may be weak as well as in premenopausal women. Thus, in the presence of estradiol, the influence of testosterone on insulin resistance may be weak. SHBG was not correlated with HOMA-IR in women receiving estrogen replacement therapy, although SHBG showed significant negative correlations with HOMA-IR in pre- postmenopausal women. The change in SHBG during only 12 months of CEE treatment may not have influenced insulin sensitivity. Further study with longer CEE treatment may be needed to determine the association of HOMA-IR with testosterone and SHBG. The present study has several limitations. First, this study was a cross-sectional design. Thus, the results of our study do not allow us to make causal inferences regarding the relationships of endocrinological hormones and SHBG with insulin resistance. Second, the sample size was small. Third, we used MPA as a progestogen with CEE. Progestogens suppress the favorable effects of estrogen. Therefore, further study on the effect of CEE alone on insulin sensitivity is needed. Finally, we assessed insulin resistance by HOMA-IR. HOMA-IR, which is a convenient assessment for insulin resistance, has been shown to have a good correlation with the euglycemic-hyperinsulinemic clamp method (27). Further studies on insulin sensitivity by using the euglycemic-hyperinsulinemic clamp procedure and glucose tolerance test may be needed. In addition, we did not examine the effects of several factors such as physical activity and dietary intake. In conclusion, the associations of sex steroid hormones with insulin resistance differ depending on the estrogen status.
